# The gastroprotective effect of ethyl acetate fraction of hot water extract of *Trichosanthes cucumerina* Linn and its underlying mechanisms

**DOI:** 10.1186/s12906-017-1796-y

**Published:** 2017-06-14

**Authors:** Ramani Karunakaran, M. Ira Thabrew, G. Mayuri Thammitiyagodage, B. Prasanna Galhena, L.D.A. Menuka Arawwawala

**Affiliations:** 1 Medical Research Institute, Dr. Danister de Silva Mawatha, Colombo, 08 Sri Lanka; 2 Institute of Biochemistry, Molecular Biology & Biotechnology, No. 90, Cumaratunga Munidasa Mawatha, Colombo, 03 Sri Lanka; 3 Department of Biochemistry & Clinical Chemistry, Faculty of Medicine, University of Kelaniya, Thallagolla Road, Ragama, Sri Lanka; 4 Industrial Technology Institute, Bauddhaloka Mawatha, Colombo, 07 Sri Lanka

**Keywords:** *Trichosanthes cucumerina* Linn, Gastroprotection, Ethyl acetate fraction, Acidity, Muscus, Antihistamine, Phytochemicals

## Abstract

**Background:**

Antacids, anticholinergic drugs, histamine H2- receptor antagonists and irreversible proton pump inhibitors have been used for the treatment of gastric ulcers. However, prolonged use of these drugs may lead to series of adverse effects such as diarrhea, headache, rash, hypertension, muscular and joint pain. Therefore, there is an urgent need of more effective and safer treatments with fewer side effects. The aim of the present study was to scientifically evaluate the gastroprotective activity of fractions of the hot water extract of *Trichosanthes cucumerina* Linn (Family: Cucurbitaceae) aerial parts with a view to identifying the fraction with the best gastroprotective activity and the possible mechanism/s by which this fraction exert gastroprotection.

**Methods:**

Gastroprotective activity of hexane fraction (HF), ethyl acetate fraction (EF), butanol fraction (BF) and aqueous fraction (AF) were evaluated by the assessment of ability to reduce the ulcer index in ethanol-induced rat model and the mode of action by which the most active fraction mediating gastroprotection.

**Results:**

EF showed the maximum gastroprotection effect followed by BF and AF. EF (75 mg/kg) exhibited significantly higher gastroprotection compared to the reference drugs. Further investigations with two lower doses of EF confirmed that EF can mediated a significant and dose dependent gastroprotection. The rats treated with the EF showed significant reduction in free acidity (45%), total acidity (by 48%) in the gastric juice, increased the amount of mucus produced by the rat gastro mucosa and potent antihistamine activity (by 25.6%). EF was also rich in phenolic compounds and flavonoids.

**Conclusion:**

Gastroprotective mechanism of EF is possibly involves inhibition of acidity, elevation in mucus content, inhibition of histamine and antioxidant mechanisms.

## Background

Gastric ulcer is a chronic/recurrent disease and currently is the most predominant gastrointestinal disease. Studies have shown that gastric ulcer occurs at least 10% of the world’s population [1, 2]. The etiology of gastric ulcers is not completely understood, however it is known that gastric lesions develop when the delicate balance between some gastroprotective and aggressive factors are lost. The major protective factors include adequate blood flow, secretion of prostaglandins, mucus and bicarbonate by resident mucosal cells. Aggressive agents include the increased secretion of hydrochloric acid and pepsin, inadequate dietary habits, free oxygen radicals, the consumption of nonsteroidal anti-inflammatory drugs and alcohol, stressful conditions and infection of *Helicobacter pylori* [2, 3]. Among the various factors, alcohol consumption is the greatest contributor to gastric ulceration [4].

*Trichosanthes cucumerina* Linn is an annual, dioecious climber belonging to the family Cucurbitaceae. It is widely distributed in Asian countries including Sri Lanka, India, Malay Penisula and Philippine [5]. The whole plant including roots, leaves, fruits, seeds have medicinal properties. The root is used as a cure for bronchitis, headache and boils. Externally, the leaf juice is rubbed over the liver to relieve liver congestion. Both the root and fruit are considered to be cathartic. The fruit is used as an anthelmintic in French Guiana. The seeds are used for stomach disorders in Malabar Coast and is also considered antifebrile and anthelmintic. The aerial parts of *T. cucumerina* are used along with other plant materials for indigestion, bilious fevers, boils, sores, skin eruptions such as urticaria, eczema, dermatitis, psoriasis diabetes and peptic ulcers [5, 6].

Studies on the pharmacological activities have shown the presence of antiinflammatory activity in root tubers [7] antidiabetic activity in seeds [8], hepatoprotective activity in whole plant [9], antidiabetic [10], anti-inflammatory [11], antioxidant [12], antibacterial [13], hypolipidaemic and antihyperglycemic activities [14] in aerial parts, and cardioprotective activity [15] in fruits of *T. cucumerina*. Further, recent scientific investigations have also shown significant gastroprotective activity in fruit seeds [16] and aerial parts [17] of *T. cucumerina* aqueous extracts. According to Arawwawala and co-workers [17], aqueous extract of aerial parts of *T. cucumerina* mediated strong gastroprotective activity against ethanol-induced gastric lesions in rats. Therefore, the aim of the present study was to evaluate the gastroprotective activity of fractions of aqueous extract of *T. cucumerina* aerial parts on ethanol induced gastric ulceration in rats, with a view to identifying the fraction that has the best gastroprotective activity, and the possible mechanism/s by which its most potent fraction exerts gastroprotection.

## Methods

### Chemicals

Absolute ethanol, Hexane, Ethyl acetate, Butanol, Diethyl Ether, Magnesium Chloride, Alcian blue, Sucrose, Acetonitrile, Phosphoric Acid, Gallic acid and Quercetin were purchased from Sigma Aldrich, Co, USA.

### Plant material

*T. cucumerina* plants were collected from Western province of Sri Lanka in between August September 2013. The plant was identified and authenticated by the curator of National Herbarium, Royal Botanical Gardens, Peradeniya, Sri Lanka. A voucher specimen (TS 02) was deposited in the Industrial Technology Institute, Colombo 7, Sri Lanka.

### Animals

Healthy adult male and female Wistar rats (weighing 200–225 g) were used throughout the experiment. They were housed individually in raised mesh bottom cages under standardized animal house conditions (room temperature: 25 ± 3 °C with 12 h dark/light cycles) and fed with standard rat feed and water ad libitum. Ethical approval for the animal experiments was obtained from the Ethical Review Committee of Medical Research Institute, Danister de Silva Mawatha, Colombo 8, Sri Lanka (Project No 34/2013). Prior to the experiments, the rats were deprived of food for 24 h, water for 12 h and kept in raised mesh bottomed cages to prevent coprophagy.

### Preparation of hot water extract (HWE)

The HWE was prepared as previously described by Arawwawala and co-workers [17]. In brief, *T. cucumerina* aerial parts were cut into small pieces and air dried. Then, 60 g of the plant material was boiled in 1.9 L of distilled water (DW) and the final volume was reduced to 240 mL by boiling over 4 h. This was repeated five times, extracts were pooled, freeze dried and stored at 4 °C until use (yield 12.5% dry weight basis).

### Fractionation of hot water extract

Freeze dried HWE (50 g) was reconstituted with DW (150 mL) and successively partitioned thrice with hexane, ethyl acetate and butanol (50 mL of each solvent). The fractions obtained were labeled as HF, EF, BF for hexane fraction, ethyl acetate fraction and butanol fraction respectively. The remaining fraction was labeled as aqueous fraction (AF). The fractions obtained were concentrated by using a rotary evaporator (Buchi, Rotavapor R − 210). Yields of the above fractions were: HF (6.8%, *w*/w), EF (15.5%, *w*/w), BF (10.6%, *w*/w) and AF (67.1%, *w*/w) respectively.

### Administration of fractions to rats

Doses of 75 mg/kg of HF, EF, BF and AF were administered orally by gastric gavage (each dose in a volume of 1 mL of DW) to separate groups of rats. The dose of 75 mg/kg corresponds to 1/10 of the dose of the HWE of aerial parts of *T. cucumerina* that showed maximum gastroprotection against ethanol- induced gastric ulcers in rats in a previous study carried out by Arawwawala and co-workers [17].

### Effects of fractions on the ulceration induced by absolute ethanol

The food and water given to rats were withdrawn for 24 h and 12 h, respectively before the commencement of the experiment. These rats were randomly divided into 7 equal groups (*n* = 8/group; 4 female rats and 4 male rats) and treated orally in the following manner: each rat in group 1 received 1 mL of DW (control group), rats in groups 2, 3, 4, 5 received 75 mg/kg of HF, EF, BF and AF in 1 mL of DW respectively while rats in group 6 received 100 mg/kg of cimetidine, a reference drug and rats in group 7 received 400 mg/kg of sucralfate, a reference drug.

After 1 h of oral treatment, each rat was given absolute ethanol (5 mL/kg) orally and kept for another 1 h. Then the rats of all groups were sacrificed after exposure to ether, stomachs were removed and inflated with 1% formalin solution and immersed in the same solution to fix the outer layer of the stomach. Each stomach was opened along the greater curvature, rinsed with tap water to remove gastric contents and blood clots followed by macroscopic determination of the gastric mucosal injury index [3, 18].

### Dose response effect of ethyl acetate fraction on the ulceration induced by absolute ethanol

Of the tested fractions, EF (at a dose of 75 mg/kg) showed maximum gastroprotection against ethanol induced gastric lesions. To evaluate whether this fraction could exert a dose response effect, investigations were also carried out to determine the gastroprotective activities of 50 mg/kg (*n* = 8/group; 4 female rats and 4 male rats) and 25 mg/kg (*n* = 8/group; 4 female rats and 4 male rats) doses of EF by method described in 2.7.

### Determination of ulcer index and percentage inhibition

Ulcer Index (UI) and percentage inhibition was calculated in ethanol induced rats as described by Nwafor et al. [19]. For the determination of Ulcer Index, the stomach was opened along the greater curvature and the inner surface was examined for ulceration with a help of a simple dissecting microscope. The stomach was examined under microscope to observe erosions and made scores as 1–5: 1 small round hemorrhagic erosion, 2- hemorrhagic erosion <1 mm, 3- hemorrhagic erosion of 2–3 mm and 5 - hemorrhagic erosion >3 mm. The score was multiplied by 2 when the width of the erosion is larger than 1 mm. The percentage of inhibition was calculated by the following formula: [(UI control –UI treated)/UI control] × 100.

### Evaluation of the mode of gastroprotective activity

The mode of action by which EF mediates its gastroprotection was assessed by determining its effects on (a) acidity and volume of the gastric juice (b) mucus content of stomach (c) anti histamine activity and (d) quantitative and qualitative evaluation of phytochemicals in the fraction.

### Evaluation of the effects on acidity and volume of the gastric juice

Sixteen rats (8 male rats and 8 female rats) were starved for 24 h and water was withdrawn for 12 h as described previously. They were randomly divided into 2 equal groups (4 male rats and 4 female rats/group). Rats in the two groups were orally treated with either 75 mg/kg of EF in 1 mL of DW or 1 mL of DW per rat. One hour later, these rats were laparotomised under ether anesthesia and at the pyloric end of the stomach was ligated with a cotton thread. The stomachs were then carefully placed back in the abdominal cavities and the rats were sutured and allowed to regain consciousness. Four hours later stomachs were removed, gastric content collected and centrifuged at 3500×*g* for 15 min for determination of gastric juice volume (mL) and pH. Acidity (total and free) in gastric secretion was determined by titration with 0.01 N NaOH according to the method described by Reitman [20].

### Determination of mucus content of stomach

Alcian blue binding to gastric wall mucus was determined as mentioned previously [17, 21]. In this experiment, 16 rats (8 male rats and 8 female rats) were starved for 24 h and water was withdrawn for 12 h as described previously. They were randomly divided into 2 equal groups (4 male rats and 4 female rats/group). Rats in the two groups were orally treated with either 75 mg/kg of EF in 1 mL of DW or 1 mL of DW per rat. One hour later, these rats were laparotomised under ether anesthesia and at the pyloric end of the stomach was ligated with a cotton thread. The stomachs were then carefully placed back in the abdominal cavities and the rats were sutured and allowed to regain consciousness. After 4 h, the rats were sacrificed with over dose of ether, each stomach was opened along the greater curvature, rinsed with 0.25 M sucrose solution. These stomachs were incubated in 10 mL aliquots of 0.1% alcian blue solution for 2 h at room temperature (30 °C). After 2 h stomachs were removed, washed with 0.25 M sucrose solution and separately incubated in 10 mL aliquots of 0.5 M magnesium chloride solution for 2 h at room temperature while shaking at 30 min intervals to elute the alcian blue bound to the mucosa of the stomachs. Two hours later, the stomachs were removed and 5 mL of each aliquot of magnesium chloride solution containing the alcian blue eluted from each stomach was shaken with 5 mL of diethyl ether. The aqueous phase was separated out, centrifuged at 3200×*g* for 5 min and the absorbance of the supernatant was measured at *λ* 605 nm. The amount of alcian blue bound per stomach in micrograms was determined using a standard calibration curve.

### Antihistamine activity

Sixteen rats (8 male rats and 8 female rats) were selected and their fur on posterior left lateral side was shaved under ether anesthesia. Twenty four hours later, these rats were randomly divided into 3 groups (4 male rats and 4 female rats/group) and treated orally in the following manner: each rat in group 1 received 75 mg/kg of EF in 1 mL of DW, rats in group 2 received chlorpheniramine, an antihistamine receptor antagonist at a dose of 0.40 mg/kg in 1 mL of DW while rats in group 3 received 1 mL of DW (control group). After 1 h, 0.05 mL of 200 μg/mL of histamine dihydrochloride was subcutaneously injected under mild ether anesthesia in the area of the skin where the fur was removed previously [22]. The radius (r) of the wheal formed was measures after 2.5 min using dial caliper and the mean value was calculated from 3 measurements for each reading. Finally, the wheal area was calculated using the formula (π = 22/7).

### Quantitative determination of total polyphenolic content

The total polyphenolic content was estimated according to the Folin – Ciocalteu method [23]. Known concentrations of EF (0.1 mL) was diluted with distilled water (0.9 mL) and mixed with 5 mL of 10 fold diluted solution of Folin – Ciocalteu reagent. Four milliliters of saturated sodium carbonate solution was added to the above mixture and shaken. The absorbance of the reaction mixture was measured at λ 765 nm after 2 h. Total phenolic content was expressed as Gallic acid equivalents (mg gallic acid/g extract).

### Quantitative determination of total flavonoid content

The total flavonoid content was determined using the Dowd method as described by Meda and co-workers [24]. In this experiment, 5 mL of 2% AlCl_3_ in methanol was mixed with the same volume of EF in known concentrations. After 10 min. The absorbance of the reaction mixture was measured at λ 415 nm. Total flavonoid content was expressed as quercetin equivalents (mg quercetin/g extract).

### Phytochemical screening

Qualitative screening of EF for alkaloids, polyphenols, flavonoids, steroids, saponins and tannins was carried out according to standard methods [25].

### Statistical analysis

Data are given as means ± S.E.M. Statistical comparisons were made using one-way ANOVA followed by Duncans multiple range test. A *P* value ≤0.05 was considered as significant.

## Results

### Effect on ethanol induced gastric lesions

**Fig. 1:**
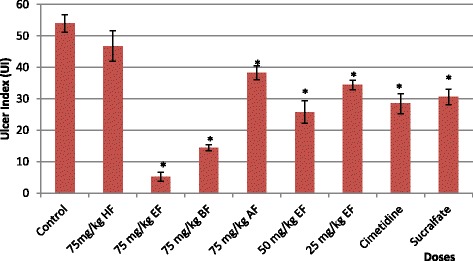
Effect of fractions of *Trichosanthes cucumerina* Linn. hot water extract, different doses of ethyl acetate fraction and reference drugs (cimetidine and sucralfate) on ulcer index in ethanol induced gastric ulcers. HF: hexane fraction, EF: ethyl acetate fraction, BF: butanol fraction, AF: aqueous fraction. Values are expressed as mean ± S.E.M., *n* = 8. * Significant when compared to the control; *P* ≤ 0.05

**Fig. 2:**
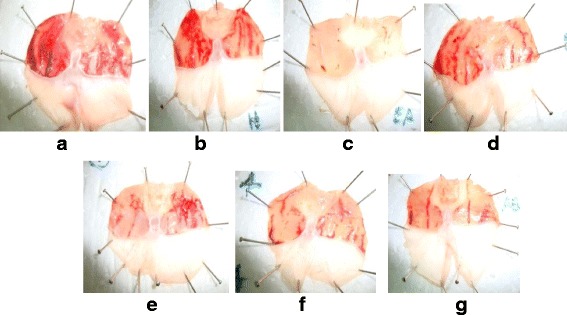
Representative macroscopic photograph of stomachs of gastric lesions induced by absolute ethanol: **a** – animals received distilled water, **b** - animals received hexane fraction, **c** – animals received ethyl acetate fraction, **d** – animals received butanol fraction, **e** – animals received aqueous fraction, **f** – animals received Sucralfate and **g** – animals received Cimetidine

Except HF all the tested fractions demonstrated significant (*P* < 0.05) gastroprotection with ulcer index score of 5.2 ± 1.5 (inhibition: 90.3%) for EF, 14.4 ± 1.2 (inhibition:73.5%) for BF and 38.3 ± 1.9 (inhibition: 29.0%) for AF (Fig. 1 & Fig. 2). Among the tested fractions, EF showed the maximum gastroprotective effect followed by BF and AF. Both EF and BF at a dose of 75 mg/kg exhibited significantly (*P* < 0.05) higher gastroprotection compared to the reference drugs, cimetidine (UI: 28.5 ± 3.6; inhibition: 47.2%) and sucralfate (UI: 30.6 ± 2.5; inhibition: 43.3%). Apart from the 75 mg/kg dose, the other two lower doses (50 mg/kg and 25 mg/kg) also showed significant (*P* < 0.05) gastroprotection compared to the control in a dose dependent (*r* ^*2*^ = 0.94) manner. Further gastroprotective effect of 50 mg/kg of EF (UI: 25.8 ± 3.6; inhibition: 52.2%) was comparable with both reference drugs while the effect of the 25 mg/kg of EF (UI: 34.4 ± 1.5; inhibition: 36.2%) was only comparable with that produced by sucralfate (Fig. 1).

### Acid secretion studies

Compared with the control, the rats treated with the EF showed significant (*P* < 0.05) reduction in free acidity (45%) and total acidity (by 48%) in the gastric juice (Table 1). Further, pH of the gastric juice increased from 3.2 to 6.1 in the treated group. However, there was no significant difference in volume of the gastric juice between the control and treated groups.

**Table 1:** Effect of ethyl acetate fraction of *Trichosanthes cucumerina* Linn. on pH, gastric juice volume, free acidity and total acidity

Groups	pH	Gastric juice volume (mL)	Free acidity (mol L^−^)	Total acidity (mol L^−^)
Control (1 mL of distilled water)	3.2 ± 0.4	3.4 ± 0.2	0.048 ± 2.7 × 10^−3^	0.079 ± 3.2 × 10^−3^
75 mg/kg of ethyl acetate fraction	6.1 ± 0.6^*^	3.5 ± 0.4	0.026 ± 2.0 × 10^–3*^	0.041 ± 3.2 × 10^–3*^

Values are expressed as mean ± S.E.M., *n* = 8

*Significant when compared to the control; *P* ≤ 0.05

### Gastric mucus studies

EF significantly (*P* < 0.05) increased the amount of mucus produced by the rat gastro mucosa (control vs treatment: 198.6 ± 9.2 vs 402.1 ± 5.8 μg/stomach).

### Antihistamine activity

EF showed potent antihistamine activity as there was a significant (*P* < 0.05) reduction (by 25.6%) in the wheal area of the rats when compared with the control group (Table 2). Further, antihistamine effect was significantly higher (*P* < 0.05) that of chlorpheniramine, the reference drug.

**Table 2:** Effect of ethyl acetate fraction of *Trichosanthes cucumerina* Linn on wheal area of the rats

Groups	Wheal area of the rats (mm^2^)
Control (1 mL of distilled water)	1.45 ± 0.08
75 mg/kg of ethyl acetate fraction	1.08 ± 0.09^*^
0.40 mg/kg of chlorpheniramine	1.22 ± 0.10^*^

Values are expressed as mean ± S.E.M., *n* = 8

*Significant when compared to the control; *P* ≤ 0.05

### Total polyphenol and flavonoid contents

The total polyphenolic content and total flavonoid content of EF was 38.82 ± 0.45 mg gallic acid equivalents/g extract and 26.30 ± 0.45 mg quercetin equivalents/g extract respectively.

### Phytochemical screening

Phytochemical screening revealed the presence of polyphenols, flavonoids, steroids and tannins in EF.

## Discussion

Plant/s based remedies have been used widely for gastroprotection by Ayurveda and Traditional medical practitioners of many Asian countries [26]. Synthetic drugs such as cimetidine, sucralfate, omeprazole are used by allopathic practitioners [27] for the treatment of gastric ulcers. Synthetic drugs have possibility of relapse of gastric ulcer, several side effects and drug interactions [27, 28]. Therefore, during the past few years, there has been an increasing interest in the development of plant based gastroprotective agents with less toxicity and side effects [26]. A previous study conducted by Arawwawala and co-workers [17] have demonstrated that HWE of *T. cucumerina* can significantly protect formation of ulcers induced by absolute ethanol without any toxic side effects [29]. In the present study, an attempt was made to investigate the gastroprotective activity of HF, EF, BF and AF obtained from *T. cucumerina* HWE with a view to identifying the most active fraction. Except HF, all the tested fractions exhibited significant (*P* < 0.05) gastroprotection. Of the active fractions, EF exhibited the maximum gastroprotective effect followed by BF and AF. Therefore, synergistic effect of the compounds present in EF, BF and AF may responsible for the gastroprotective activity of the HWE of *T. cucumerina.* Both EF and BF exhibited significantly (*P* < 0.05) higher gastroprotection compared to the reference drugs, cimetidine and sucralfate.

Administration of absolute ethanol by gavage has long been used as a reproducible method to induce gastric injury in experimental animals [30]. Ethanol has been proposed to be responsible for the stimulation of gastric acid secretion, through reflex release of gastrin and histamine from sensitive nerve terminals present in the gastric mucosa [31]. In the present investigation, EF showed significant (*P* < 0.05) reduction in free acidity (45%) and total acidity (by 48%) and elevation in pH of the gastric juice in the treated group. Moreover, EF showed potent antihistamine activity and the effect was significantly higher (*P* < 0.05) that of chlorpheniramine, the reference drug. It is well established that antihistamine drugs like cimetidine which block H_2_ receptors in the stomach, reduce the acidity of gastric juice [32]. Therefore, the reduction in acidity of gastric juice mediated by the EF may also be attributable to its antihistamine effect. Although, H_2_ receptor blockers are also expected to reduce the secretion of gastric juice [32] such an effect was not observed in the present study. The reason for this discrepancy is not clear. However, similar effects have also been demonstrated by extracts of some other plants such as *Curatella americana* [33] and *Zanthoxylum rhoifolium* [34].

Mucus is an important protection factor of the gastric mucosa. It presents itself as a transparent gel formed by water and glycoproteins, which covers and protects the gastrointestinal mucosa against irritating agents, such as ethanol and HCl [34]. Ethanol also causes severe gastric mucosal ulceration either by acting directly on the gastric mucosa, altering protective factors or indirectly by increasing the release of vasoactive products such as histamine from mast cells [30]. It was observed that EF significantly (*P* < 0.05) increased the amount of mucus produced in the rat gastro mucosa by 102%. This is important because the success of the gastric ulcer treatment relies not only on the blockage of acid secretion, but also on the augmentation of the protective factors of the gastric mucosa. The mucus also plays a role in keeping the mucosa pH near neutral and protecting it from the action of the gastric acid [35]. Further prostaglandins production in large amounts by the gastrointestinal mucosa can prevent by ulcerogens [36]. In the present study, when the ulcer lesions are induced by absolute ethanol, the cytoprotective effect of an antiulcer agent can be mediated through endogeneous prostaglandin production [36, 37]. Therefore, EF may stimulate the secretion of prostaglandins or possess prostaglandin like substances. In our studies, inhibition of acidity (both total and free) and increase of mucus content equally contributed to the gastroprotction mediated by EF. Similar mode of action has also been reported in some other plant extracts such as bark extract of *Rhizophora mangle* [38], fruit extract of *Piper tuberculatum* [39], leaves extract of *Copaifera langsdorffii* [2].

*T. cucumerina* is proven to have significant anti-inflammatory activity and the effect was comparable with the reference drug, indomethacin which is a NSAID [11]. The major mechanism by which NSAIDs exert anti-inflammatory effects is by inhibiting the cyclooxygenase (COX) pathway that produces prostaglandins, thromboxanes and prostacyclin. COX exists in two isoforms, COX - 1 and COX – 2. Inhibition of COX – 2, is responsible for the anti-inflammatory effects of NSAIDs. On the other hand, inhibition of COX - 1 by these agents causes damage to the gastrointestinal tract [40, 41]. It is well established that cucurbitacins can inhibit the COX pathway [42] and *T. cucumerina* also contain several cucurbatacins [6]. In the present study, high gastroprotective activity was exerted by EF suggests that COX – 2 inhibitory activity of *T. cucumerina* may be greater than that of the COX - 1 inhibitory activity because they produced marked gastroprotection against ethanol induced gastric ulceration. Furthermore, β – sitosterol, a triterpenoid is proven to have potent gastroprotection [43] and *T .cucumerina* also contains β – sitosterol [6]. Therefore, presence of β – sitosterol may also contribute for potent gastroprotection of *T.cucumerina.*

Gastric ulcer disease is a multi-factorial disease and the significant role played by reactive oxygen species and free radicals during its pathogenesis is well experimented in both human and experimental animals [44]. Phenolic and flavonoid compounds have been shown to be responsible for the scavenging reactive oxygen species and free radicals. EF was rich in high content of phenolic and favonoid compounds. Therefore, this may be one of the mechanisms by which EF mediates its gastroprotection. Phenolic compounds such as allylpyrocatechol, curcumin, gallic acid, quercetin, resveratrol, polyphenols of pomegranate, green tea, grapes and apple are known to exhibit potent gastroprotection by different mechanisms such as (a) ability to prevent gastric mucosal damage (b) inhibitory effect of *H. pylori* infection (c) enhancement of bicarbonate and mucus secretion (d) acceleration of angiogenesis and mucosal blood floor and (e) antisecretory activity [45, 46]. Tannins have also been shown to exert antiulcerogenic actions due to their protein precipitating and vasoconstricting effects [47]. Their astringent action can help precipitate microproteins on the ulcer site, thereby forming an impervious layer over the lining that hinders gut secretions and protects the underlying mucosa from toxic and other irritants [19, 48]. Therefore, presence of tannins may also contribute to the gastroprotion mediated by EF. Furthermore, EA did not produce any signs of hepatotoxicity (levels of AST, ALT, ALP) or renotoxicity (levels of creatinine and urea) in terms of liver function and kidney function assessments or unacceptable hematological effects in terms of RBC, WBC, differential WBC, % PCV, MCV, MCH, MCHC and Hb concentration (unpublished data). Therefore, EA at the dose of 75 mg/kg can be safely used for therapeutic purposes.

## Conclusions

In conclusion, present study has revealed for the first time, that among the fractions separated from the HWE of *T. cucumerina,* the best gastroprotective effect in ethanol- induced rat model is exhibited by EF. The gastroprotective mechanism of EF is multi-factorial and possibly involves (a) inhibition of acidity (both total and free) (b) elevation in mucus content (c) inhibition of histamine and (d) antioxidant mechanisms. Further studies are necessary to isolate the active compound/s present in the EF with a view to developing an agent that could be used in clinical practice for the management of gastric ulcers.

## Abbreviations

AF: Aqueous fraction
ALP: Alkaline phosphatase
ALT: Alanine aminotransferase
AST: Serum aspartate aminotransferase
BF: Butanol fraction
COX: Cyclooxygenase
DW: Distilled water
EF: Ethyl acetate fraction
HF: Hexane fraction
HWE: Hot water extract
MCH: Mean cell hemoglobin
MCHC: Mean cell hemoglobin concentration
MCV: Mean cell volume
NSAID: Non steroidal antiinflammatory drug
PCV: Packed cell volume
RBC: red Blood cells
WBC: White blood cells
